# Mental health predictors of Internet gaming disorder: a longitudinal study

**DOI:** 10.47626/1516-4446-2024-3816

**Published:** 2025-01-22

**Authors:** Guilherme Borges, Ricardo Orozco, Raúl Gutierrez-Garcia, Yesica Albor, Ana Lucía Jiménez Pérez, Karla Patrica Valdés-García, Patricia M. Baez Mansur, María Anabell Covarrubias Díaz-Couder, Corina Benjet

**Affiliations:** 1 Centro de Investigación en Salud Mental Global, Instituto Nacional de Psiquiatría Ramón de la Fuente Muñiz, Ciudad de México, México; 2 Facultad de Estudios Superiores, Universidad De La Salle Bajío, Salamanca, México; 3 Universidad Autónoma de Baja California, Campus Ensenada, Baja California, México; 4 Facultad de Psicología, Universidad Autónoma de Coahuila, Saltillo, México; 5 Universidad La Salle Victoria, Ciudad Victoria, México; 6 Coordinación de Investigación, Universidad la Salle Noroeste, Ciudad Obregón, México

**Keywords:** Internet gaming disorder, anxiety, depression, substance use disorders, longitudinal

## Abstract

**Objective:**

We investigated whether a wide range of baseline mental disorders among university students predict Internet gaming disorder (IGD) 1 to 3 years later.

**Methods:**

This prospective cohort study was conducted in six Mexican universities and had a 1- to 3-year follow-up period (September 2018 to June 2022). Participants were 1st-year university students (n=2,144) free of symptoms indicative of IGD at entry (baseline). Ten mental disorders (bipolar, major depressive disorder, generalized anxiety disorder, panic disorder, alcohol use disorder, drug use disorder, binging and/or purging, intermittent explosive disorder, psychotic experiences, and attention deficit hyperactivity disorder) at baseline were investigated as risk factors for IGD at the end of the follow-up. We used Cox regression to model the IGD incidence rate.

**Results:**

Any baseline mental disorder was associated with a 2.33 times (1.26-4.31) higher rate of IGD 1 to 3 years later. Several individual disorders were associated with rates of IGD in multiple models, with comorbid conditions diminishing most of these associations.

**Conclusion:**

Only major depressive disorder and bipolar disorder remained associated with new cases of IGD. The discrepant results of longitudinal studies on the role of specific mental disorders in the development of IGD should be further investigated.

## Introduction

While Internet gaming disorder (IGD)^1^,^2^ has been clinically defined for more than 10 years, we still know little about its mental health determinants. When the DSM-5 and ICD-11 were published, evidence about the impact of mental disorders on IGD development was scant, with few longitudinal studies available.^3^-^5^ The original 2013 formulation and the new DSM-5TR^6^ describe IGD as associated with major depression, attention deficit hyperactivity disorder (ADHD) and obsessive-compulsive disorder. The ICD-11 states that GD “commonly co-occurs with disorders due to substance use, mood disorders, anxiety or fear-related disorders, attention deficit hyperactivity disorder, obsessive-compulsive disorder, and sleep-wake disorders.”^7^

Most of what we know about the co-occurrence of IGD with other mental disorders comes from cross-sectional studies on the clinical features of IGD and its prevalence and correlates.^8^ These correlates have been recently grouped as “(i) gaming-related factors (i.e., structural characteristics), (ii) individual factors (i.e., person-based characteristics), and (iii) environmental factors (i.e., situational characteristics).”^9^ We focus here on the role of mental health disorders as individual determinants of new-onset IGD, for which longitudinal studies that can separate the causes and consequences of IGD are less common.^10^ Because some reviews did not differentiate longitudinal from cross-sectional studies, their estimates on the role of mental disorders as risk factors for IGD are more prone to bias.^11^,^12^ A comprehensive review of longitudinal studies on IGD identified only six studies with data on anxiety, four with data on attention/hyperactivity, and 12 with data on depression, with generally weak effect sizes.^13^ Another review identified only six longitudinal studies on ADHD and GD.^14^ As expected, not all longitudinal reports agree on the likelihood that mental disorders lead to IGD.^12^,^15^-^17^ Differences in methods, screening scales, analytical procedures, age groups, and follow-up periods may explain the inconsistent results. Most literature reviews lack longitudinal studies that include a wide range of mental disorders as possible predictors of IGD, as classified in the DSM-5 and ICD-11, as well as controls for comorbidity.

We report here on a longitudinal cohort of Mexican college students from six universities located across the country. We investigated the longitudinal associations of a large list of mental disorders with new-onset IGD. These disorders include bipolar disorder, major depressive disorder, generalized anxiety disorder, panic disorder, alcohol use disorder, drug use disorder, binging and/or purging, intermittent explosive disorder, psychotic experiences, and ADHD.

## Methods

### Study design

This prospective cohort study included a follow-up period of 1 to 3 years.^18^ Not all students responded each year, so the follow-up for some was 1, 2, or 3 years. Exposed participants were 1st-year university students, free of IGD at entry (baseline), who reported baseline symptoms compatible with a diagnosis of bipolar disorder, major depressive disorder, generalized anxiety disorder, panic disorder, alcohol use disorder, drug use disorder, binging and/or purging, intermittent explosive disorder, psychotic experiences, or ADHD. These baseline disorders were investigated as potential risk factors for IGD incidence at the end of the follow-up.

### Sample and procedures

The participants were a cohort of 1st-year university students enrolled in six Mexican universities (two public and four private) for the PUERTAS study (University Project for Healthy Students),^19^ which consisted of a baseline web-based survey conducted in the 2018-2019 academic year and three follow-up surveys ending in 2022. These students were the first cohort in which an IGD scale was administered.^20^ All 1st-year students aged 18 or older were eligible (n=11,099) and were recruited through school events (e.g., new student orientation and 1st-year courses), in which a URL to the online survey was provided. In 2018, 8,122 students agreed to participate in the baseline survey, with a 73% overall response rate. Later, e-mail invitations to three follow-up surveys were sent at the beginning of each academic year, with 31.8% of the respondents completing at least one questionnaire. Data collection started in September 2018 and ended in June 2022, resulting in a total of 2,267 students with complete data on the main variables for this analysis (see the Strengthening the Reporting of Observational Studies in Epidemiology flowchart in Supplementary Figure S1). The PUERTAS study is part of the World Health Organization World Mental Health International College Student initiative.^21^ Participation was confidential and voluntary, requiring informed consent.

### Measures

The online, self-report survey was developed for the World Mental Health International College Student initiative^21^ and comprises the well-validated measures described below.

#### Baseline control variables

The sociodemographic variables consisted of sex (“male,” “not male”) and age. Fully adjusted models also included whether students slept at least 8 hours for ≤ 2 days/week or > 2 days/week.^22^

#### Outcome variable: Internet gaming disorder during follow-up

All participants were asked whether they played video games in the past 12 months. The full IGD scale was applied only to gamers who screened positive for the following: if they played, on average, at least 1 day per week (during weekdays or weekends) for ≥ 30 minutes (henceforth, “active gamers”). IGD was assessed in a section consisting of 23 items covering the nine symptoms (domains) described in the DSM-5, as formulated by an international consensus led by Nancy Petry, which included an English version and a Spanish translation.^20^,^23^ As per DSM-5 guidelines, the presence of five of nine symptoms is considered probable IGD. A description of each IGD symptom, together with the psychometric properties of the scale, has been reported elsewhere^20^; briefly, the scale’s reliability was adequate (α = 0.816), and the factor analysis fit indices were excellent (comparative fit index = 0.994, Tucker Lewis index = 0.992, root mean square error of approximation = 0.030). All students classified as positive for IGD at baseline (n=123) were removed from the follow-up analysis, while those negative for IGD at baseline (n=2,144) were followed up for new-onset cases.

#### Mental health disorders as exposure variables at baseline

The Composite International Diagnostic Interview Screening Scales^24^ were applied at baseline to assess DSM diagnostic criteria for lifetime symptoms of mood disorders: bipolar I and major depressive disorder, anxiety disorders (generalized anxiety disorder and panic disorder), and substance use disorders (drug use disorder). They are called screening scales because they are non-clinician diagnosed, self-administered self-report measures. However, they do assess each of the diagnostic criteria for the aforementioned disorders. These scales agree with blinded clinical diagnoses, having an area under the curve range of 0.70-0.78.^24^ The AUDIT screening scale^25^ was used to assess lifetime alcohol disorder and agrees with clinical diagnosis, having an area under the curve range of 0.78-0.91.^26^ We also screened for lifetime eating problems (binging and/or purging), intermittent explosive disorder, and psychotic experiences. Six-month ADHD was evaluated with the Adult ADHD Self‐Report Scale.^27^ Although self‐report scale results cannot be considered a clinical diagnosis of these disorders, they do indicate clinically significant symptoms.

#### Covariates

Variables considered in the adjusted analysis were male sex (yes/no), age at baseline, and sleeping ≥ 8 hours a night. A summary measure of any disorder was also included in the analyses. Additionally, the models were fully adjusted for possible comorbidities and included all 10 mental disorders.

### Statistical analysis

This prospective cohort study assessed the effect of mental disorders at baseline (exposure) through the first occurrence (i.e., incidence) of IGD (outcome) during follow-up.^28^ For this, we used data from all students who completed at least two measurements and, as mentioned previously, we excluded 123 students who were IGD-positive at baseline.

Incidence rates per 1,000 persons per year were estimated by dividing the number of incident IGD cases over the sum of person-years (per 1,000). For censored observations (those who did not develop IGD), person-time was computed as the difference between the date of the last follow-up and baseline. We followed a similar procedure for incident IGD cases, but discounted half of the time between incident occurrence and the previous follow-up date.

Crude and adjusted hazard ratios and their respective 95%CI were estimated for IGD incidence by fitting proportional-hazards Cox models.^29^ First, we fitted crude models for each mental disorder and for the summary variable of any mental disorder. We then adjusted each model by sex, baseline age, and sleep duration. To account for comorbidities, we fitted a single model in which all 10 mental disorders were entered simultaneously.^9^ Finally, to obtain a more parsimonious multivariate model for mental disorders, we used recommendations based on change in mean squared error^30^ in which age, sex, and sleep duration were selected as key confounding variables. A nonresponse weight by sex and age was computed to account for losses to follow-up^31^ (Supplementary Table S1), and it was incorporated in all Cox models.^32^ Statistical tests were evaluated at an alpha level of 0.05 and all data analyses were performed in Stata 18.0.^33^

### Ethics statement

This study was approved by the National Institute of Psychiatry in Mexico Research Ethics Committee.

## Results

At baseline 31% of the IGD-negative and 78% of the IGD-positive participants were men (Table 1). Those with IGD were also more likely to report few hours of sleep. Importantly, apart from drug abuse/dependence, all other disorders were more prevalent among those with IGD at baseline, with 88.6% of the IGD-positive participants reporting one of the 10 mental disorders included here. It was common for IGD to persist: of the 123 IGD cases detected at baseline, 44 (35.8%) reported IGD in the following 1 to 3 years.

As per our methodology, the 123 baseline cases of IGD were discarded and the remaining 2,144 IGD-negative students were followed up to detect new cases of IGD during the subsequent 1 to 3 years. Table 2 presents the distribution of these 80 incident IGD cases according to the 10 baseline mental disorders and the summary measure of any disorder. At baseline, the incidence rate among those with any mental disorder (Table 2, bottom) was 22.09 per 1,000 person-years of IGD, while those free of any disorder had a much lower IGD incidence rate: 9.26 per 1,000 person-years. This table shows that, except for panic disorder, the incidence rate of IGD was higher among those with than among those without a mental disorder. For example, the IGD incidence rate was 45.92 and 16.92 per 1,000 person-years among those who did and did not report drug abuse/dependence at baseline, respectively.

**Figure 1:**
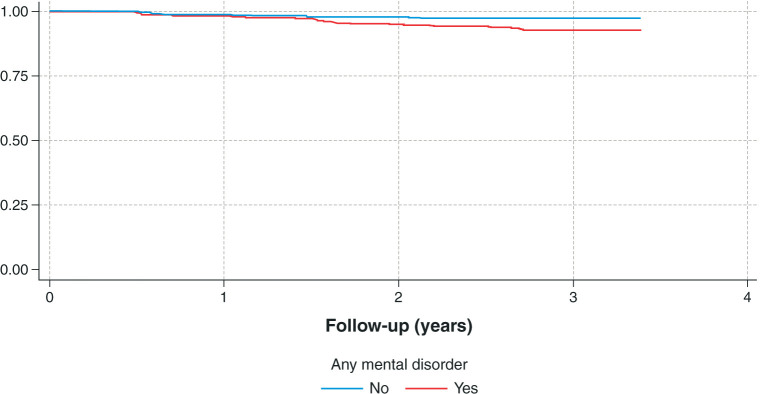
Co-occurrence of Internet gaming disorder and any mental disorder. Kaplan-Meier survival curves. Weighted data.

We modeled this follow-up data through Cox regression analyses, the results of which are shown in Table 3. Crude bivariate analyses showed that major depressive disorder, bipolar I disorder, drug abuse/dependence, binging/purging, intermittent explosive disorder, and psychotic experiences all increased the likelihood of developing IGD. Except for panic disorder, the other disorders also increased the rate of IGD, although not significantly. These basic results did not change in multivariate models that controlled for age, sex, and sleep duration. In the multiple models, having any disorder was associated with a 2.33 times greater likelihood of developing IGD. The Kaplan Meier curve for any disorder (Figure 1) suggests that after the 1st year of follow-up, the IGD survival curves began to differ for those with and without any mental disorder.

As shown in Table 4, the full multivariate model, including demographics and all mental disorders, shows decreases in all individual estimates except panic disorder. This indicates that at least part of the increase in IGD rates in individual disorders is due to comorbid conditions. We then fitted a more parsimonious model including the most important individual mental disorders, as described in the methods section. In this final model (Table 4), the mental disorders that predicted IGD were major depressive disorder (HR = 1.87) and bipolar disorder (HR = 2.70).

## Discussion

In summary, we found that persistent IGD during 3 years of follow-up was common (35.8%). Having a mental disorder at baseline increased the likelihood of IGD during follow-up by 2.33 times. While most individual mental disorders increased the rate of IGD, comorbidity explained most of these longitudinal effects. In our multiple models, only major depressive disorder and bipolar disorder remained associated with IGD incidence.

A meta-analysis of nine longitudinal studies published until 2017 estimated that 50% of IGD cases were persistent, despite great heterogeneity (with estimates ranging from 84 to 24%).^34^ Two more recent studies estimated IGD persistency at 39.4% over 2 years among 4th- and 7th-graders in South Korea^35^ and at 41.7% among six junior high schools in Shanghai, China.^36^ We estimate that approximately one in three university respondents with IGD at baseline would still report it after 1 to 3 years, which is in the lower range of these reports. The IGC incidence in our sample of university students, who are older than the populations of similar studies, strongly suggests that IGD is not a transitory disorder that resolves in adulthood. However, due to the small number of persistent cases in our sample, we could not explore the determinants of persistency, which is a subject for future research.

In our sample, comorbidity between IGD and other mental disorders was the rule, with almost 89% of IGD cases reporting at least one of the 10 other included mental disorders. As evident from meta-analysis,^8^,^10^,^13^,^14^ most studies on mental health disorders focus on the role of depression as a likely determinant of IGD, followed by studies on anxiety and others on ADHD. Few studies have included more than two disorders, and we know of no other study that has investigated a large number of disorders and has included a summary measure of “any” mental disorder, as presented here. The result of adjusting for any mental disorder resulted in an overall decrease in the hazard ratios for most individual disorders, which shows the extent of comorbidity in IGD. Nevertheless, most longitudinal studies ignore comorbidity when modeling and presenting the results for a single disorder. Depression and bipolar disorder remained firmly associated with IGD onset in our data, suggesting that they are a greater driving force than impulsivity or compulsivity on IDG incidence. This supports the self-medication hypothesis of the disorder’s etiology,^9^ as well as the finding of a Mexican study that in comorbid mood and substance use disorders, the onset of mood disorders generally precedes that of substance use disorders.^37^ Alternatively, it is possible that a particular disorder, such as depression or bipolar disorder, affects IGD incidence through a general psychopathology dimension, called the p factor.^38^,^39^ In fact, in a recent study that tested different theories of the p factor, bipolar disorder, mania, and personality disorders (not included in our current study) yielded the highest loading on the p factor.^40^ Thus, when we controlled for comorbidity, the fact that bipolar disorder was one of two disorders to remain significant could indicate that a general propensity to psychopathology is associated with IGD onset rather than any particular disorder. However, the existence of a common unknown cause for both psychopathology and IGD is also possible.^15^

Our survey has significant limitations to consider. Because the follow-up response rate was low, we weighted the data to adjust for non-response bias regarding sex and age. However, the sample could have been biased in other ways. Although both public and private universities were included, they were not randomly selected, meaning that study participants are not representative of all university students in Mexico.

Despite its limitations, this study provides evidence about the effects of a long list of common mental disorders on IGD incidence among students, which goes beyond traditional samples of unknown gamers from gaming websites. Our 3-year follow-up period exceeded the usual single academic year used in most studies. Our longitudinal approach provided stronger evidence for directionality and causality than correlational studies, while controlling for mental health symptoms at baseline allowed us to determine the effects of comorbid disorders. The conflicting results of longitudinal studies on the role of particular mental disorders, such as depression or bipolar disorder, warrants further research.

In conclusion, our longitudinal study contributes to the scant literature on IGD among young adults, going beyond cross-sectional studies on the short-term (1 year) impact of mental health problems on IGD. Our analysis suggests that only a few mental disorders have an independent effect on IGD incidence. Further research is needed on comorbidity between IGD and other mental disorders and should be explored longitudinally.

## Disclosure

The authors report no conflicts of interest.

## Figures and Tables

**Table 1:** Baseline sociodemographic characteristics and psychiatric disorders according to the presence of Internet gaming disorder in the last 12 months (n=2,267)

	IGD scale score ≥ 5 in the last 12 months	
	No	Yes
	n=2,144 (94.6)	n=123 (5.4)
Male		
No	1,480 (69.0)	27 (22.0)
Yes	664 (31.0)	96 (78.0)
Age group		
18-19 years	1,673 (78.0)	92 (74.8)
≥ 20 years	471 (22.0)	31 (25.2)
Sleeps ≥ 8 hours		
≤ 2 days/week	852 (39.7)	64 (52.0)
> 2 days/week	1,292 (60.3)	59 (48.0)
Major depressive disorder		
No	1,847 (86.1)	97 (78.9)
Yes	297 (13.9)	26 (21.1)
Bipolar I disorder		
No	2,075 (96.8)	117 (95.1)
Yes	69 (3.2)	6 (4.9)
Panic disorder		
No	2,065 (96.3)	114 (92.7)
Yes	79 (3.7)	9 (7.3)
Generalized anxiety disorder		
No	2,102 (98.0)	119 (96.7)
Yes	42 (2.0)	4 (3.3)
Probable alcohol dependence		
No	2,076 (96.8)	114 (92.7)
Yes	68 (3.2)	9 (7.3)
Drug abuse/dependence		
No	2,040 (95.1)	119 (96.7)
Yes	104 (4.9)	4 (3.3)
Binging and/or purging (screening items)		
No	1,475 (68.8)	70 (56.9)
Yes	669 (31.2)	53 (43.1)
Intermittent explosive disorder (screening item)		
No	1,413 (65.9)	60 (48.8)
Yes	731 (34.1)	63 (51.2)
Psychotic experiences (screening items)		
No	1,773 (82.7)	82 (66.7)
Yes	371 (17.3)	41 (33.3)
Possible ADHD (in the last 6 months)		
No	1,370 (63.9)	37 (30.1)
Yes	774 (36.1)	86 (69.9)
Any mental disorder		
No	749 (34.9)	14 (11.4)
Yes	1,395 (65.1)	109 (88.6)
IGD scale score ≥ 5 during follow-up		
No	2,064 (96.3)	79 (64.2)
Yes	80 (3.7)	44 (35.8)

Data presented as n (%).

ADHD = attention deficit hyperactivity disorder; IGD = Internet gaming disorder.

All disorders are lifetime, except ADHD (in the 6 months prior to baseline) and IGD (in the last 12 months). Unweighted data.

**Table 2:** Sample sizes, incident cases, and incidence rates for IGD according to mental disorders at baseline (n=2,144)

	n	Incident IGD cases	Person-years	IR per 1,000
Major depressive disorder				
No	1,847	62	3,936.6	15.75
Yes	297	18	625.2	28.79
Bipolar I disorder				
No	2,075	75	4,427.1	16.94
Yes	69	5	134.7	37.13
Panic disorder				
No	2,065	79	4,394.9	17.98
Yes	79	1	166.9	5.99
Generalized anxiety disorder				
No	2,102	77	4,468.8	17.23
Yes	42	3	93.0	32.27
Probable alcohol dependence				
No	2,076	75	4,435.8	16.91
Yes	68	5	126.0	39.68
Drug abuse/dependence				
No	2,040	71	4,365.8	16.26
Yes	104	9	196.0	45.92
Binging and/or purging (screening items)				
No	1,475	47	3,152.7	14.91
Yes	669	33	1,409.0	23.42
Intermittent explosive disorder (screening item)				
No	1,413	43	3,010.0	14.29
Yes	731	37	1,551.7	23.84
Psychotic experiences (screening items)				
No	1,773	56	3,808.0	14.71
Yes	371	24	753.8	31.84
Possible ADHD (in the last 6 months)				
No	1,370	43	2,934.5	14.65
Yes	774	37	1,627.3	22.74
Any mental disorder				
No	749	15	1,619.2	9.26
Yes	1,395	65	2,942.6	22.09

ADHD = attention deficit hyperactivity disorder; IGD = Internet gaming disorder; IR = incidence rate.

All disorders are lifetime, except ADHD (in the last 6 months) and IGD (in the last 12 months). The IR was calculated among those free of DSM-5 IGD at baseline. Unweighted data.

**Table 3:** Baseline mental disorders as predictors of DSM-5 IGD during follow-up - bivariate Cox models adjusted by sex, age, and sleep duration

	Crude models			Models (adjusted by sex, age, and sleep)		
	HR	p-value	95%CI	HR	p-value	95%CI
Major depressive disorder						
No	1.00			1.00		
Yes	1.72*	0.049	1.00-2.95	1.98*	0.020	1.11-3.54
Bipolar I disorder						
No	1.00			1.00		
Yes	2.62*	0.037	1.06-6.48	2.60*	0.035	1.07-6.32
Panic disorder						
No	1.00			1.00		
Yes	0.37	0.324	0.05-2.65	0.36	0.303	0.05-2.51
Generalized anxiety disorder						
No	1.00			1.00		
Yes	1.44	0.544	0.45-4.63	1.55	0.469	0.47-5.08
Probable alcohol dependence						
No	1.00			1.00		
Yes	2.19	0.097	0.87-5.52	1.92	0.171	0.75-4.88
Drug abuse/dependence						
No	1.00			1.00		
Yes	2.83*	0.004	1.39-5.80	2.60*	0.010	1.26-5.38
Binging and/or purging (screening items)						
No	1.00			1.00		
Yes	1.69*	0.024	1.07-2.68	1.83*	0.012	1.14-2.95
Intermittent explosive disorder (screening items)						
No	1.00			1.00		
Yes	1.59*	0.044	1.01-2.50	1.52	0.076	0.96-2.41
Psychotic experiences (screening items)						
No	1.00			1.00		
Yes	2.20*	0.002	1.34-3.59	2.04*	0.005	1.24-3.37
Possible ADHD (in the last 6 months)						
No	1.00			1.00		
Yes	1.49	0.085	0.95-2.34	1.42	0.140	0.89-2.24
Any mental disorder						
No	1.00			1.00		
Yes	2.42*	0.003	1.34-4.35	2.33*	0.007	1.26-4.31

ADHD = attention deficit hyperactivity disorder; HR = hazard ratios; IGD = Internet gaming disorder.

HR estimated with weighted Cox models. All disorders are lifetime, except ADHD (in the last 6 months) and IGD (in the last 12 months). Incident DSM-5 IGD is the outcome in all models.

* p < 0.05.

**Table 4:** Baseline mental disorders as predictors of DSM-5 IGD at follow-up - multivariable Cox models

	Full model			Multiple model		
	HR	p-value	95%CI	HR	p-value	95%CI
Major depressive disorder						
No	1.00			1.00		
Yes	1.83	0.063	0.97-3.46	1.87*	0.042	1.02-3.42
Bipolar I disorder						
No	1.00			1.00		
Yes	2.48	0.071	0.93-6.62	2.70*	0.042	1.04-7.02
Panic disorder						
No	1.00			1.00		
Yes	0.19	0.081	0.03-1.23	0.20	0.087	0.03-1.26
Generalized anxiety disorder						
No	1.00					
Yes	1.08	0.916	0.26-4.57			
Probable alcohol dependence						
No	1.00					
Yes	1.25	0.679	0.44-3.57			
Drug abuse/dependence						
No	1.00					
Yes	1.85	0.138	0.82-4.14			
Binging and/or purging (screening items)						
No	1.00			1.00		
Yes	1.41	0.229	0.80-2.48	1.43	0.199	0.83-2.49
Intermittent explosive disorder (screening item)						
No	1.00			1.00		
Yes	1.09	0.734	0.65-1.84	1.15	0.580	0.70-1.91
Psychotic experiences (screening items)						
No	1.00			1.00		
Yes	1.64	0.106	0.90-2.99	1.71	0.069	0.96-3.04
Possible ADHD (6-month)						
No	1.00					
Yes	0.96	0.887	0.57-1.63			

ADHD = attention deficit hyperactivity disorder; HR = hazard ratios; IGD = Internet gaming disorder.

HR estimated with weighted Cox models. All disorders are lifetime, except ADHD (in the last 6 months) and IGD (in the last 12 months). Incident DSM-5 IGD was the outcome in all models. Multiple and full models add controls for baseline sex, age, and sleep.

* p < 0.05.
