# Supplementary material for: Mental health predictors of Internet gaming disorder: a longitudinal study
Source: Braz J Psychiatry. 2025 Jan 22;47:e20243816. doi: 10.47626/1516-4446-2024-3816 (PMC12679694; doi:10.47626/1516-4446-2024-3816)
Supplement: Supplementary file 1 [file bjp-47-e20243816-suppl1.pdf]

http://doi.org/10.47626/1516-4446-2024-3816

Supplementary Figure S1 STROBE flowchart

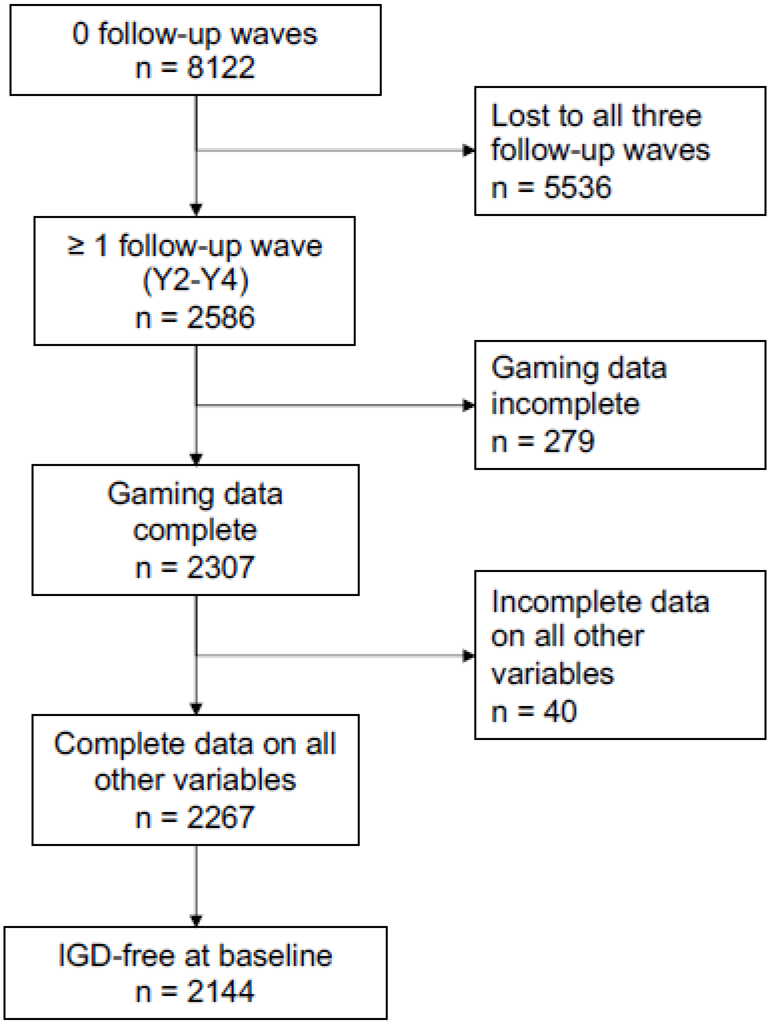

IGD = Internet Gaming Disorder.

http://doi.org/10.47626/1516-4446-2024-3816

Supplementary Table S1 Comparison of baseline characteristics among included participants and those lost to follow-up or with incomplete data (n=8,122)

	**Sample for IGD-incidence complete data, all variables**		
	**Lost to follow-up/ incomplete data n=5,855 (72.1%)**	**≥ 1 follow-up/ complete data n=2,267 (27.9%)**	**Test**
Male sex			
No	2,921 (50.4%)	1,507 (66.5%)	<0.001
Yes	2,876 (49.6%)	760 (33.5%)	
Age group			
18-19 years	4,163 (71.1%)	1,765 (77.9%)	<0.001
≥ 20 years	1,692 (28.9%)	502 (22.1%)	
Sleeps ≥ 8 hours			
≤ 2 days/week	2,256 (39.0%)	916 (40.4%)	0.236
> 2 days/week	3,533 (61.0%)	1,351 (59.6%)	
Major depressive disorder			
No	5,246 (89.6%)	1,944 (85.8%)	<0.001
Yes	609 (10.4%)	323 (14.2%)	
Bipolar I disorder			
No	5,647 (96.4%)	2,192 (96.7%)	0.590
Yes	208 (3.6%)	75 (3.3%)	
Panic disorder			
No	5,642 (96.4%)	2,179 (96.1%)	0.602
Yes	213 (3.6%)	88 (3.9%)	
Generalized anxiety disorder			
No	5,767 (98.5%)	2,221 (98.0%)	0.095
Yes	88 (1.5%)	46 (2.0%)	
Probable alcohol dependence			
No	5,592 (95.5%)	2,190 (96.6%)	0.027
Yes	263 (4.5%)	77 (3.4%)	
Drug abuse/dependence			
No	5,571 (95.1%)	2,159 (95.2%)	0.870
Yes	284 (4.9%)	108 (4.8%)	
Binging and/or purging (screening items)			
No	4,259 (72.7%)	1,545 (68.2%)	<0.001
Yes	1,596 (27.3%)	722 (31.8%)	
Intermittent explosive disorder (screening item)			
No	3,984 (68.0%)	1,473 (65.0%)	0.008
Yes	1,871 (32.0%)	794 (35.0%)	
Psychosis (screening items)			
No	4,985 (85.1%)	1,855 (81.8%)	<0.001
Yes	870 (14.9%)	412 (18.2%)	

http://doi.org/10.47626/1516-4446-2024-3816

Possible ADHD (6-month)			
No	3,829 (65.4%)	1,407 (62.1%)	0.005
Yes	2,026 (34.6%)	860 (37.9%)	
Any mental disorder			
No	2,243 (38.3%)	763 (33.7%)	<0.001
Yes	3,612 (61.7%)	1,504 (66.3%)	

IGD = Internet gaming disorder.
Some frequencies do not add up to the total sample size due to missing values.
p-values from chi-squared tests.
